# Decentralization of the provision of health services to people living with HIV/AIDS in rural China: the case of three counties

**DOI:** 10.1186/1478-4505-9-9

**Published:** 2011-02-11

**Authors:** Xiulan Zhang, Pierre Miège, Yurong Zhang

**Affiliations:** 1 School of Social Development and Public Policy, Beijing Normal University, New Main Building Room 2011, 19 Xin Jie Kou Wai Street, Beijing 100875, PR China

## Abstract

This study is based on a large-scale household survey and in-depth interviews of key informants that was conducted in villages in three counties of two provinces in China. We assess the new decentralized service provision system for people living with HIV/AIDS in rural populations in China. Since 2003, new social assistance schemes, and, more importantly, decentralization of routine treatment and care to community health stations, were progressively implemented in rural areas most affected by the HIV/AIDS epidemic. Though some problems remain, such as persistent discrimination towards infected patients and the lack of sufficient training of medical staff, the new decentralized pattern of service provision has lowered barriers to health access and alleviated economic pressure on affected households.

## Introduction

The HIV epidemic has been accelerating in China since the mid 1990 s. While by October 2009, 319,877 cases had been reported since the first case in 1986 [1], a joint mission by World Health Organization (WHO) and the Chinese Ministry of Health (MoH), UNAIDS estimated the number of people living with HIV/AIDS (PLWHA) to be 740,000 [2]. China is classed as having a concentrated epidemic with a low HIV prevalence, (around 0.05 to 0.08%, depending on data source), with high incidence rates among specific sub-populations. Indeed, 39% of reported HIV-positive cases have been transmitted through needle exchange by injecting drug users (IDUs), 19% through blood sales, 18% through heterosexual transmissions, and 1% through homosexual transmission. In total 70% of PLWHA are between 20-39 years old, and over 70% are male [3].

A specificity of the HIV/AIDS epidemic in China is its higher prevalence in rural areas [4], which have been especially affected by the changes in the public health system in the last two decades. The public health system has undergone profound transformations since the launch of economic reforms in the late 1970 s, which, among other consequences, weakened access to health for rural populations [5,6]. Until the 1990 s, there were more health facilities per capita in the countryside than in the cities, but recently this was reversed and the gap has steadily expanded. For example, studies have shown that in 2005, there were 2.6 health workers for 1,000 people in the rural areas compared to 6.2 in urban ones [7]. In 2002, more than 80% of rural households had no health insurance [8,9]. Until the mid-2000 s, lack of health insurance and rising cost of care kept a large proportion of the rural population outside the health care system [10,11]. From the early 2000 s, new reforms of the public health system have been progressively implemented in order to address these inequalities in access to health services [8]. At the same time, the acceleration of the HIV/AIDS epidemic in the last decade has led the government to design specific policies to address the needs of PLWHA in the rural areas, especially through a new social and financial support scheme and a decentralization of the service provision system.

The official response to the HIV/AIDS epidemic has been progressive, and started to take momentum after 2000. The State Council Coordination Conference Mechanism for HIV/AIDS Prevention and Control was created in 1996, and the following year the government published the first "Medium-and Long-Term Programs for the Prevention and Control of AID", which mostly organized the effort in terms of prevention and surveillance mechanisms of the epidemic. From 2001, as the number of infected persons was rising, a series of regulations were introduced to address the problem of access to treatment: the first "Action Plan for HIV/AIDS Prevention and Control in China (2001-2005)" called for the creation of a community-based network of services for the prevention and treatment of HIV/AIDS, including the provision of medical care to those already infected. In 2003-2004, two major policies were set up. First, with the support of the Global Fund, the first comprehensive treatment program, the China CARES (China Comprehensive AIDS RESponse), started to provide Antiretroviral (ARV) in 51 high prevalence mostly rural counties, and was rapidly extended in the following years, providing treatment for 36,000 PLWHA in 1,190 counties in 2008 [3]. Second, in order to address the needs of economically vulnerable PLWHA, the "Four Frees and One Care" program was launched in 2004. This scheme targets rural and poor urban residents, providing free treatment, free drugs to HIV-infected pregnant women, free voluntary HIV screening tests, and free education for orphans of people living with HIV/AIDS. "Care" takes the form of financial support to PLWHA households [12]. Through a remarkable increase in the State funds, with already almost 944 million yuans ($US 10 million) in 2007 [3], matched with local funds dedicated to this purpose, new innovative prevention programs were designed targeting high-risk groups living in rural areas such as injection drug users and commercial sex workers. More importantly, locally managed financial support schemes for rural households of PLWHA have been set up. These have played a crucial role in reducing the financial difficulties of families affected by HIV/AIDS and in strengthening the social integration of PLWHA [12].

In terms of access to treatment and care, in its "Regulations on AIDS Prevention and Treatment" and in "China's Action Plan for Reducing and Preventing the Spread of HIV/AIDS (2006 - 2010)", both issued in 2006 [13], China has chosen to implement at the county and village level a decentralized pattern favoring community-based care and provision of free Antiretroviral Therapy (ART) - as well as partially subsidized opportunistic infections (OI) treatment [14]. In this system, private doctors in the villages, many being former "barefoot doctors", are charged with missions that supplement health stations, especially to visit patients and perform small procedures such as injections [15].

This new health provision system set up in rural China has not been unnoticed and sometimes praised [16], but no study has tried to evaluate its performance. Health care provision for HIV infected patients living in rural China has not been thoroughly researched, partly because of the diversity of local situations. The studies available present either one case [17], or the situation in a single province [6,18]. In this paper, we present results from preliminary research conducted in three rural villages, situated in two provinces. Though limited in scope, this study, based on both a household questionnaire and in-depth interviews, suggests that community-level services have greatly expanded access to health for PLWHA in relatively poor rural counties and have enabled a better follow-up of patients. This study also highlights changes that need to be implemented in order to further improve such services.

## Methods

This study is part of a larger research project which includes a large-scale household survey aimed at understanding the impact of different pathologies, including HIV/AIDS, on social and economic conditions of rural households. In June 2008, a team of a researcher and two assistants conducted in-depth interviews of key informants, focusing on treatment and care of PLWHA. A total of 21 persons, including managers of health care facilities, health providers and patients, were interviewed. Interviews were based on a semi-structured questionnaire, which included a set of questions adapted to position and function of the persons interviewed. Each interview lasted approximately one and half hour.

In this research, health provision refers to the distribution of ARV and OI treatment for HIV patients, as well as testing facilities and hospitalization. Health service providers include doctors and nurses in clinics and hospitals, as well as village private doctors, usually former "barefoot doctors". The latter were usually trained for two to three years after junior-high school, and receive on an average less than a day of continuing training every year [16].

This study was conducted in communities located in three counties of two provinces, Henan and Anhui Provinces, both seriously affected by HIV/AIDS epidemic, with most of PLWHA infected through blood selling in the first half of the 1990 s. The research sites include Wenlou AIDS village clinics in Shangcai county (Henan province), Huanzhuang community health station in the Economic and Technological Development Zone (hereafter ETDZ) located in Fuyang municipality (Anhui province) and Caisi community service station in Funan county, also part of Fuyang municipality. Shangcai county hosted in 2002 the first pilot program for free ART distribution in China, and provided training sessions for most health providers [19]. This county is representative of the early implementation of the policy of community-based treatment and care of HIV patients in high-prevalence counties. Funan county and ETDZ belong to the same city, Fuyang (Anhui province), but differ in that ETDZ is a mixed area, with rural households and new factories and companies, located very close to Fuyang city center, whereas Funan is more rural and situated further away from the urban core of Fuyang.

The health facilities in these three sites are tasked to provide adequate treatment and care to a relatively large number of patients, as indicated in table 1. Figures were provided by the different health facilities in the three sites. It must be noted that the decision to start treatment is based mainly on health checks realized at the service stations, due to lack of equipments to perform CD4 counts.

**Table 1:** Total number of persons living with HIV/AIDS as reported by health services in the three villages

Research sites	Number of PLWHA
Huanzhuang community service station (ETDZ, Fuyang municipality, Anhui)	312

Caisi community service station (Funan county, Anhui)	209

Wenlou village clinics (Shangcai county, Henan)	365

In these three counties, most contaminations were the consequence of blood selling. Donors received money to donate blood; In order to increase donation frequency, in most collection centers blood was pooled in machines to extract plasma. In many cases, sellers were re-injected with blood after the plasmapheresis, leading to considerable HIV transmission [20]. This mode of contamination has led, starting in 1996, to policy responses from central and local authorities, especially the setting up of China CARES and of the "Four Frees and One Care". Such HIV patients face less discrimination from the community than drug users. Therefore, these three counties do not fully reflect the situation of other counties where most contaminations are due to drug use or sexual transmission. Nevertheless, the findings are useful as they suggest an improved access to health services for PLWHA since the decentralization of treatment and care to the community-level.

This paper analyses findings about HIV/AIDS from a larger project studying the organization of health care services in rural China for a large range of pathologies. One limitation of this specific study of HIV/AIDS related health services is that it focuses on the organization of these services and the problems that still limit access to treatment. It does not assess the drug regimen nor other aspects of the treatment.

## Results

### Health provision system for HIV/AIDS and OI treatment and care

In order to provide timely and adequate health services for patients, health provisions for PLWHA have been organized at the village- and township-level, with community health stations established in places with high HIV/AIDS prevalence rates. Usually, doctors and nurses working in these community facilities are sent from higher health institutions. Furthermore, ETDZ and Shangcai officially implement a "referral model", by referring patients who need more complex treatment and care to designated higher-level hospitals (either at the county- or municipal-level). In Funan, however, the policy is for such higher-level hospitals to send experienced and trained staff to the community health stations in order to diagnose and treat complex cases. According to doctors interviewed, there is no regular policy about the number and training of medical staff transferred from higher institutions to the village clinics. When this research was conducted in one village of this county, Caisi, two doctors had been sent for one year from the county-level hospital to the community health station. As a village clinic doctor explained: "there is no need to refer patients to the county hospital. They can receive the same treatment standards here as in the county hospital."

For this study, it has not been possible to collect coherent data on patients under treatment at the village level. In table 2, we therefore present data collected during the fieldwork in 2008 concerning the three counties to which the villages studied belong. While differences exist among counties, these figures still provide valuable information about the institutions where patients receive treatment. An interesting element is that in Funan and Shangcai counties, despite differences in their referral systems, almost the same proportion of patients are referred to the county level hospital (table 2). This suggests that in fact local doctors eventually refer a comparable percentage of patients to higher-level health institutions, raising questions about the usefulness of sending urban doctors to rural clinics.

**Table 2:** Health institutions where patients do receive treatment

		Village Clinic	Township Clinic	County Hospital	County CDC	Municipal-level Hospital	Total
Shangcai county	n	191	10	45	5	6	257
	
	%	74.3%	3.9%	17.5%	1.9%	2.3%	100%

Funan county	n	28	11	8	0	2	49
	
	%	57.1%	22.4%	16.3%	.0%	4.1%	100%

ETDZ	n	10	17	4	0	32	63
	
	%	15.9%	27.0%	6.3%	.0%	50.8%	100%

**Total**	n	**229**	**38**	**57**	**5**	**40**	**369**
	
	%	**62.1%**	**10.3%**	**15.4%**	**1.4%**	**10.8%**	**100%**

In all three sites, most patients are taken care of at community-based health facilities. In Shangcai county, the most rural of the three, almost three quarters of patients receive treatment at the village level. Funan county is more urbanized and more people live in the township. Therefore, when village and township health institutions are added up, almost 80% of patients receive treatment at the community level. The situation is different in ETDZ where only half of patients are taken care of at the community level. As it is a district located nearby Fuyang urban core, and therefore very close to its municipal-level hospital for infectious diseases, some HIV patients choose to go directly to this latter facility; Others are referred to this hospital when the community-level structures cannot provide adequate services.

In all three sites, interviews with PLWhA and service providers suggest that village private doctors play an important role in supervising patients' health conditions and their adherence to treatment, especially in Caisi community health station (Funan county). The tasks of private doctors include the provision of basic services (such as injections and basic health checks) at the community health station under the guidance of doctors sent from county-level hospitals. They also follow-up patients, by visiting them in their house two to three times a month, and monitor their adherence to treatment.

### The cost of ARV and OI treatment has been lowered but in some cases remains an obstacle

In the three counties studied, PLWHA can benefit from free ARV treatment through the "Four Frees and One Care" scheme. However, OI treatment and care are not covered by the latter system, and in the three sites surveyed such costs are covered by insurance schemes which vary. Depending on the type of scheme to which each patient and his/her household is entitled, between 10 and 15% of the cost of OI treatment remains at their charge. Doctors and managers of health institutions interviewed stress that it is important to let patients pay for part of their treatment in order to prevent abuse of the system. As a manager states: "Patients are required to pay 10% to 15% of their medicine cost mainly to avoid them coming for drugs every day, as would be the case with a completely free policy."

Except in Funan county, free HIV testing is not universal: almost 39% of patients in Shangcai and 10% in ETDZ had to pay for this test. It must also be noted that in those three sites, as it is the case in most of the country, no second-line regimen is included in the free access to treatment schemes. Finally, when patients are hospitalized, extra costs, such as the fees for hospitalization, basic health tests and the payment of meals, which are of the responsibility of the patient, only add up.

Therefore, patients interviewed acknowledge that the introduction of free access to ARV treatment, and the reimbursement of a large part of health expenses, have greatly lowered the financial burden for PLWHA households and expanded access to health for HIV-infected. But some patients, especially those facing more difficult personal and economic situations, report having difficulties accessing all the treatment and care they would need.

### Lack of medical and testing equipment at the community level and short supply of some

Currently, national health authorities have decided that both ARV and OI treatment mainly rely on drugs produced domestically. Due to constrained industrial and technological capabilities, there are regular shortages of treatment. As one patient complains: "In the past, we could use any drugs we needed. But now a lot of drugs are in short supply. There are shortages of even some of the most basic medicine."

In addition, the list of the ARV drugs prescribed within the "free four one care" scheme is not sufficiently up-to-date, and does not include a second-line, affecting patients who need to move to a second-line regimen. Doctors interviewed suggest that experts should update the list of prescribed drugs more frequently in order to improve the quality of treatment.

Many doctors and patients interviewed also stress the lack of medical equipment in the health stations, especially for routine blood testing of patients. This influences the quality of treatment and care provided to PLWHA, as the decision to start ARV treatment is generally based on superficial health checks realized by insufficiently experienced doctors. As one doctor acknowledges: "We give the same treatment when symptoms look similar. We are unable to make further checks to determine what causes the symptoms. (...) Here, we have not received enough professional training and we lack relevant medical equipment and a laboratory. If one treatment method does not work, we try an alternative one."

### Insufficient training of some health-care professionals and discriminations as barriers to access to treatment

This study could not investigate more thoroughly the types of training received by the different service providers, but the problem of insufficient experience and training, especially at the community-level, was often reported by patients and acknowledged by some doctors interviewed. Indeed, if in the three sites surveyed, village private doctors play a central role, as they follow HIV patients and supervise daily treatment and care, most of them (between 60% and 70% depending on the surveyed sites) had been "barefoot doctors", and therefore have received only limited medical training. Consequently, they are facing great challenges in providing care to PLWHA, in supervising their adherence to treatment and in helping them with treatment side effects.

Even among those medically-trained, interviewees say that they are sometimes insufficiently prepared to take care of co-infections at the later stage of the disease. As a doctor from a county-level hospital explains: "Currently, the provision of ARV treatment is mature, and it is not relevant to continue making it the main topic of our professional training. Meanwhile, as a large group of patients are now entering more advanced stages of the disease, the treatment of opportunistic infections is more complex and challenging, and medical staff in the community health stations are in urgent need of training in this area."

As already explained, in Funan doctors are regularly sent from county-level hospital to the community health stations. But during our fieldwork in Cause (Funan county), the two transferred doctors had no specialized training concerning HIV epidemic; one had actually been sent there in order to be able to prepare some medical exams in a quieter environment. Discussion and exchange of experience with the local village doctors were limited. Such problems seriously weaken the quality of treatment and care provided at the community-level.

In all three sites, some patients interviewed reported having been refused non-HIV related treatments by medical staff, for example in orthopedics, gastroenterology and gynecology departments. Some HIV/AIDS patients have also been denied non-disposable medical equipment, even when they urgently needed such devices. This problem can be related to the lack of awareness and training about HIV/AIDS epidemic among some doctors outside of epidemiological departments who appear to sometimes discriminate HIV patients by refusing them access to some treatment and operations.

## Discussion

This research highlights the diversity of local arrangements under the official policy of decentralization of treatment and care at the community level. The three selected counties do not illustrate the whole HIV epidemic in China, as most HIV patients were contaminated through blood selling. The interviews and observations from our research project provide valuable information filling a knowledge gap concerning the way the official policy of decentralization of treatment and care to the village/community level has had an impact on access to health care and treatment for PLWHA in rural China. This paper represents the first such study, and therefore, despite its limitations, contributes to our understanding on the latest reforms of the Chinese health care system.

In Funan and Shangcai counties, the vast majority of patients (almost 80%) have access to testing and to treatment at the community-level, either at the village or township clinics depending on where they live. In ETDZ, situated close to the city-center and the municipal-level hospital for infectious diseases, around half of patients access treatment at this hospital. In all three sites, however, daily care and follow-up of patients is the responsibility of village private doctors and community health stations.

The interviews conducted for this research suggest that the decentralization of HIV/AIDS treatment and care at the community level, implemented mainly since 2003, has proved effective in reaching rural infected patients, who had been marginalized by the reforms of the health system since the 1980 s. Previously, other studies had stressed long distances and costly transportation to explain the low access to health in some rural areas [21]. In the three sites studied, designated community-level health facilities are always located relatively close to the populations covered, eliminating therefore a potential barrier to access to health care.

However, problems remain, and some doctors interviewed highlighted a persisting lack of adherence to treatment which, though improving, remains an important preoccupation, as other studies have confirmed [22-24]. Another problem suggested in interviews of both patients and doctors is the cost of treatment, a proportion of which is still expected to be met by patients. In the three sites studied here, such costs are relatively lower than had been observed a few years ago [6], but remain an obstacle for many patients. This is particularly true as hospitalization and some of the routine tests are not included in reimbursement schemes.

Interviews also suggest that discrimination and fear of exposure within the medical community represent major obstacles to access health care services for PLWHA. Many studies have documented this problem [25,26]. Medical personnel might show a perception of occupational risk [27], or a fear of being discriminated against by the community if they treat HIV patients [28,29], both factors explaining their refusal to treat HIV patients. Our interviews suggest that medical staff need some additional training, in order to help them better understand HIV epidemic and risks. They also suggest that health facilities should provide personnel with all the necessary health safety equipment and procedures, for example disposable needles, disposable gloves, etc., which would reduce the fear of infection among doctors and nurses [30].

More generally, both health providers and patients interviewed highlighted the need for more training of medical staff, particularly to village private doctors. In the three sites studied, most of these private doctors were former "barefoot" doctors. They are extremely committed and in practice are the real foundations of the HIV-related health provision system in the countryside. They know most of the inhabitants of their village, and visit all patients every week, checking their health condition, their adherence to treatment, and providing advice on diet and ways to cope with the side effects of ARV and OI treatment. As they are at the core of the rural provision system, they should also benefit the most from training on such issues as HIV treatment, adherence problems and ways to help patients cope with side effects. Interviews also suggest that county- and municipal-level hospitals doctors and nurses should receive additional training, and the medical personnel sent to the community health stations must be selected according to their experience.

## Competing interests

The authors declare that they have no competing interests.

## Authors' contributions

PM helped designing the questionnaire and participated in the analysis. ZX and PM wrote the first draft of the manuscript. ZY participated in the questionnaire design, survey and analysis. All authors read and approved the final manuscript.
